# Case Report: Efficiency of Embolization Microcoils for the Repair of Brachiocephalic Vein Perforation During Hemodialysis Catheter Placement

**DOI:** 10.3389/fmed.2021.726120

**Published:** 2021-09-17

**Authors:** Ying Hu, Fujia Gu, Ping Yuan, Min Shi, Liang Ma, Yan Zha, Ping Fu

**Affiliations:** ^1^Department of Nephrology, Kidney Research Institute, West China Hospital of Sichuan University, Chengdu, China; ^2^Department of Nephrology, People's Hospital of Guizhou Province, Guiyang, China; ^3^Department of Intervention, People's Hospital of Guizhou Province, Guiyang, China; ^4^Department of Vascular Surgery, People's Hospital of Guizhou Province, Guiyang, China

**Keywords:** brachiocephalic vein perforation, hemodialysis cuff catheter, embolization micro coils, chest repairing, case report

## Abstract

**Background:** The cuff catheter is one of the most common routes of vascular access in hemodialysis patients, while severe complications can occur during cuff catheter placement, such as bleeding, hematoma, and artery or vein damage. During catheterization, brachiocephalic vein perforation associated with a mediastinal lesion is rare. Open chest repair is effective for brachiocephalic vein perforation during catheter placement, but it entails a risk of potentially lethal trauma. Interventional treatment can be considered to reduce injury in this context, but relevant reports are limited.

**Case report:** Herein, we describe our experience with a 68-year-old male hemodialyzed patient in whom cuff catheter vascular access was required for regular hemodialysis. He complained of mild pain in the left side of his chest during cuff catheter placement. The surgeon immediately checked the location of the catheter. Digital subtraction angiography revealed that the hemodialysis cuff catheter had punctured the mediastinal area from the left brachiocephalic vein. The patient was diagnosed with left brachiocephalic vein perforation (d ≈ 5 mm). Fortunately, the brachiocephalic vein perforation was successfully repaired with two embolization microcoils after comprehensive assessment and multidisciplinary consultation.

**Conclusion:** Brachiocephalic vein perforation can be repaired with embolization microcoils during hemodialysis catheter placement, and this method of interventional treatment is safe and effective.

## Introduction

The cuff catheter is used for either temporary or permanent vascular access in hemodialysis patients. Although the use of these catheters is beneficial, there are several potential complications that should be borne in mind during catheterization or hemodialysis. Of these, iatrogenic perforation of the brachiocephalic vein is one of the most dangerous because it can lead to massive hemorrhage, hemorrhagic shock, and even death. The most common treatments for brachiocephalic vein perforation were surgical repair and endovascular stent grafting in the past, both of which entail a risk of potentially lethal trauma. It was recently reported that innominate vein perforation with pericardial effusion was successfully treated ***via*** coil and glue embolization of the sinus tract under the guidance of digital subtraction angiography (DSA) (1), but such reports are limited. Herein, we describe a case of left brachiocephalic vein perforation into the mediastinum with no hematoma formation that was successfully treated solely with an embolization coil and review the case in terms of diagnosis, treatment, and related reports.

## Case Report

**Figure 1:**
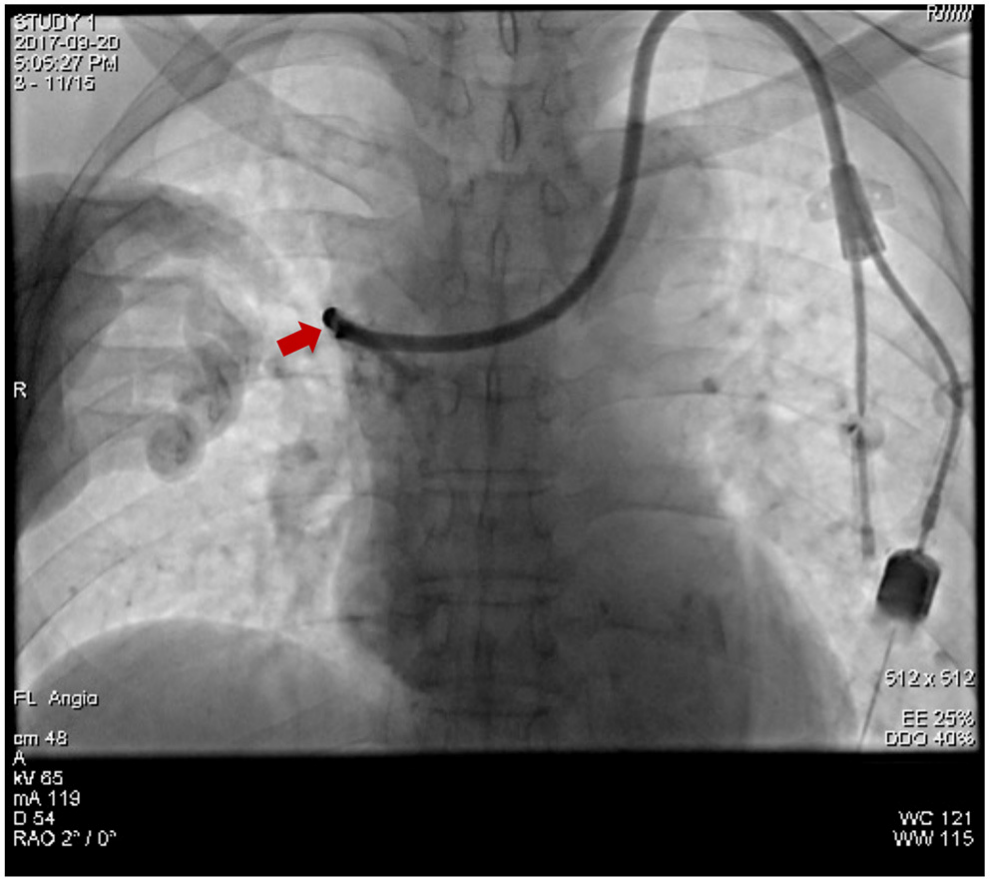
The tip of the hemodialysis cuff catheter could not reach the upper 1/3 of the right atrial position.

The patient was a 68-year-old man with a 30-year history of hypertension and regular hemodialysis for 11 years who had undergone forearm arteriovenous fistula surgery to treat end-stage renal disease 9 years prior. A long-term hemodialysis catheter (14.5F/23 cm, BARD) had been placed in the right internal jugular vein for hemodialysis 2 years prior. 2 days before the current visit, the hemodialysis cuff catheter ports caused blood oozing during hemodialysis, and the right cuff catheter was pulled out to stop the blood oozing. 1 day before the current visit, a cuff catheter was placed in the patient's left internal jugular vein for hemodialysis. Using the Seldinger puncture technique, the operation was performed under DSA in an interventional radiology room. The cuff catheter was introduced *via* smooth introduction and positioning of the guidewires. After dilator and guidewire removal, the catheter was inserted into the left internal jugular vein with the removal of the peel-away sheaths (2). The patient reported mild pain in the left side of his chest during catheterization. The surgeon immediately checked the location of the catheter placement and found that there was no venous blood being drawn from the cuff catheter. DSA analysis revealed that the tip of the catheter could not reach the upper 1/3 of the right atrium (the tip of the catheter was in this position, which can meet the requirements of hemodialysis, as shown in Figure 1). The surgeon decided to adjust the catheter the next day because the patient could not continue to tolerate the operation.

**Figure 2:**
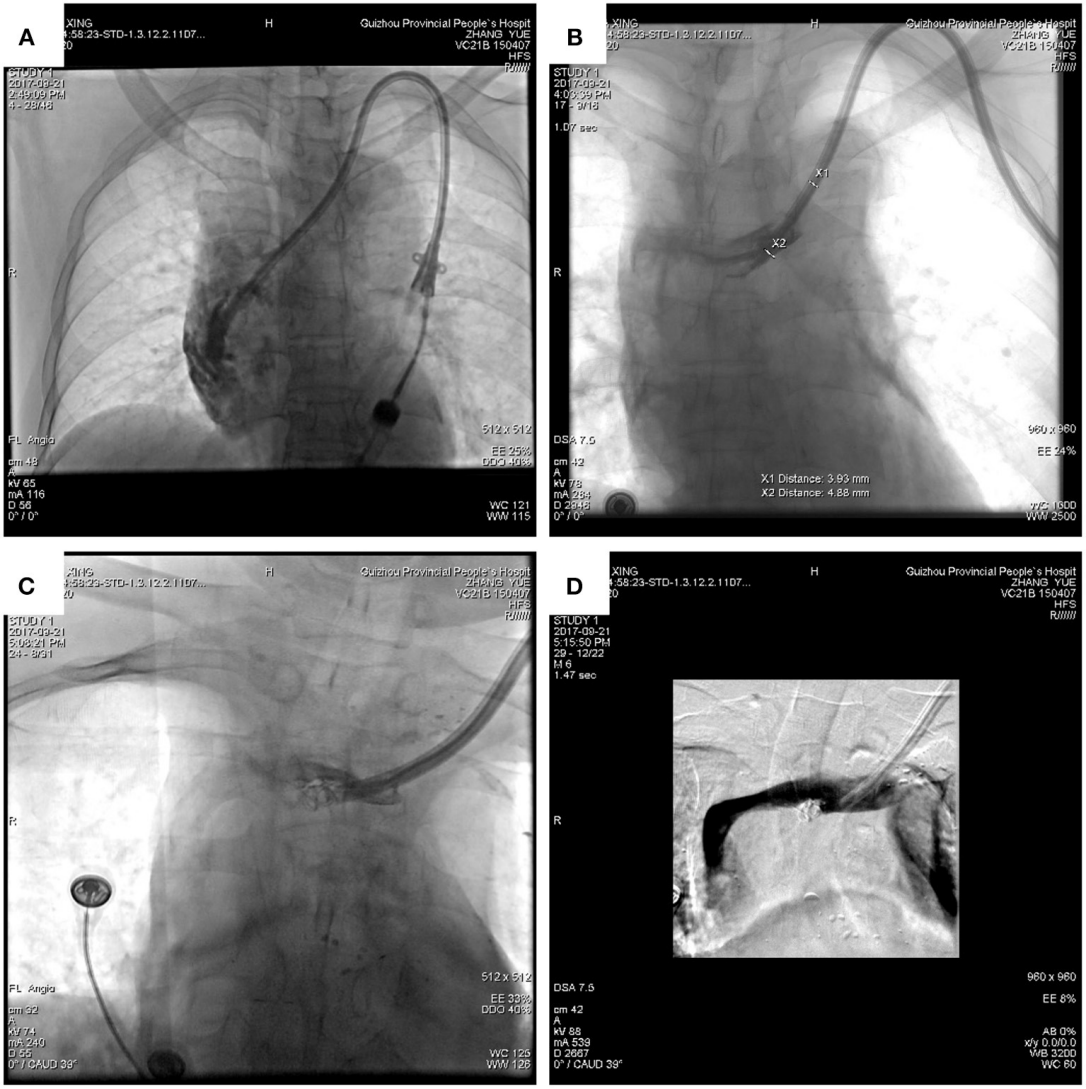
DSA revealed that the hemodialysis catheter had penetrated the brachiocephalic vein into the mediastinum **(A)**. The diameter of the vascular rupture was approximately 5 mm **(B)**. The catheter was drawn out slowly, and the vascular rupture was blocked with two embolization coils **(C)**. The catheter was returned to the lumen of the brachiocephalic vein, the perforation was blocked, and there was no bleeding **(D)**.

The next day attempts were made to adjust the catheter *via* a rotating method, but during that process, the catheter pierced the brachiocephalic vein and entered the mediastinum (Figure 2A). Opening the breast area and suturing the brachiocephalic vein were initially considered, but it was surmised that this could be lethal given the patient's history of long-term anemia and uremia. Immediate multidisciplinary consultation with interventional, vascular surgery, and cardiology experts was undertaken, and two main courses of action were considered. One was to repair the rupture, retain the cuff catheter, and continue the hemodialysis treatment at the same time. The other was not to perform an open chest operation and consider alternatives. It was concluded that the patient could not endure further trauma because he was weak.

**Figure 3:**
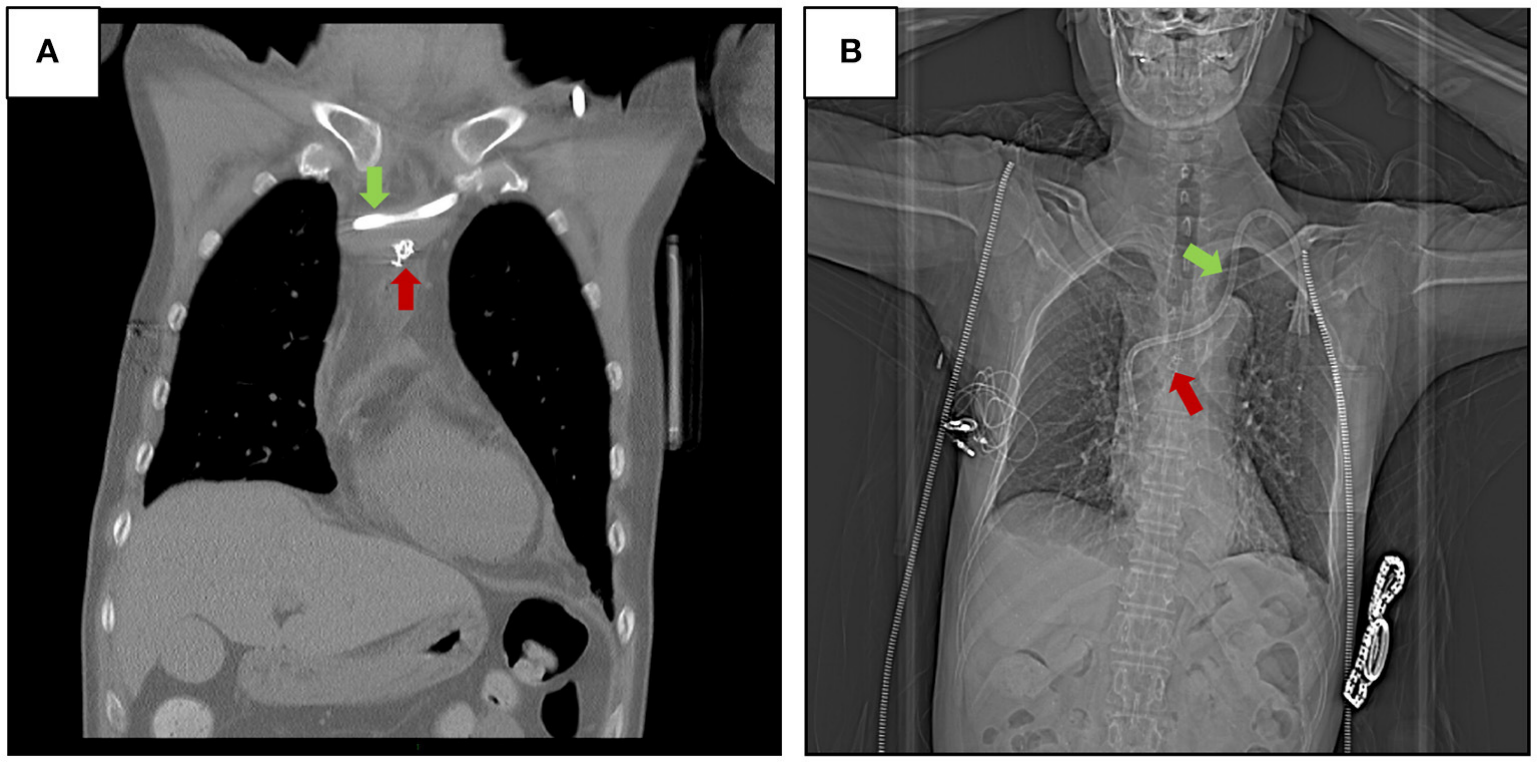
Two embolization microcoils (COOK d ≈ 5 mm) were inserted to block the vascular rupture **(A)**, and X-ray revealed that the cuff catheter and embolization microcoil were appropriately located in the brachiocephalic vein **(B)**.

Because the patient had no hematoma formation and the rupture was small (d ≈ 5 mm; Figure 2B), and based on the experience of interventional doctors, an embolization microcoil was used to fix the perforation with the assistance of a vascular surgeon (Figures 2C,D). This minimized the potential for injury. It has been reported that embolization microcoil is usually used to block bleeding arteries (3), and that this treatment is less traumatic than other methods. The surgeon used this method to perform femoral vein puncture on the right lower limb of the current patient, inserted an ordinary catheter (4F cobra) through the guide wire into the vascular access point, and measured the diameter of the blood vessel rupture under DSA. The diameter of the vascular rupture was approximately 5 mm, so the surgeon chose a d ≈ 5 mm embolization microcoil (COOK) to insert into the catheter to block the vascular rupture. When the first embolization coil was inserted, it drifted with the blood flow. The surgeon then put another embolization microcoil into it. While observing the vascular rupture again, the surgeon could not see any contrast agent flowing out from the vascular rupture. This indicated that the closure was successful, and the patient reported no discomfort. Blood pressure and heart rate were normal. The surgeon adjusted the catheter again and put it into the normal position, and the empty needle could draw out venous blood from the catheter smoothly and without resistance. The catheter was successfully replaced as shown in Figure 3A. The patient could then continue to undergo hemodialysis *via* the left cuff catheter (Figure 3B).

## Discussion

The cuff catheter is an important vascular access route in hemodialysis patients, especially those with blood vessels in poor condition. The present case confirmed that hemodialysis cuff catheters can cause vascular injury. An improperly performed operation could lead to serious complications, such as hematoma, pneumothorax, a misplaced catheter, and thyroid neck stem injury (3), but simple damage to the vein is rare. In order to avoid this kind of complication, ultrasound-guided and DSA approaches are recommended (4). Ultrasound-guided catheter placement reduces the occurrence of puncture-associated complications (5), but its usefulness is limited in life-threatening cases. At deep locations, the catheter may not be detected *via* ultrasound guidance, and this may delay diagnosis and treatment. In the current case, because the left cuff catheter was placed under DSA in the interventional radiology room, the brachiocephalic vein perforation was readily detected and diagnosed. This prevented further development of hematoma. The present case suggested that left cuff catheter insertion should be performed directly under DSA. Multidisciplinary expert consultation was also indispensable after the complication occurred in the current patient, and it could facilitate determination of the most appropriate treatment strategy comparatively rapidly. The complication was treated in a timely manner, and the patient's trauma was minimized. Relevant reports suggest that several factors can increase the risk of central venous perforation (Table 1). The distance from the left internal jugular vein to the atrium is longer than the right internal jugular vein. Venous perforation occurs more frequently with left intubation, especially left subclavian intubation. In the cases listed in Table 1, there are 5 cases of venous perforation in the left side and 4 cases of venous perforation in the left subclavian. Pua (10) and Tilak et al. (11) reported that more complications were associated with the left side due to the angulations of vessels involved in left vs. right insertions. The anatomical location in the current patient indicates that the operation of left cuff catheters should be performed under DSA to reduce the risk of complications. The experience of the surgeon is also a key factor. Many serious complications have occurred involving junior doctors. To reduce the likelihood of complications, junior doctors need to practice the operation under the guidance of senior doctors. Lastly, preoperative assessment is another important factor, especially in elderly patients who have been diagnosed with cardiovascular and cerebrovascular diseases. Such conditions can complicate the surgery.

**Table 1:** The related literatures of venous perforation during the hemodialysis catheter placement (*n* = 7).

**Case report**	**Age/y**	**Sex**	**Catheter type**	**Intubation position**	**Complications**	**Treatment**	**Outcome**
Chow et al. (6)	66	Male	Temporary catheter	Left internal jugular vein	Catheter guidewire in the head and neck	Surgical intervention	Recover
Cooley et al. (7)	52	Female	Cuff catheter	Left subclavian vein	Pulmonary artery injury	Paramedian sternotomy	Recover
Li et al. (5)	50	Female	Temporary catheter	Left subclavian vein	Subclavian vein perforated	Thrombin injection with balloon dilatation	Recover
Siordia et al. (8)	75	Female	Cuff catheter	Left subclavian vein	Innominate vein perforation	A partial upper sternotomy	Recover
Johari et al. (9)	39	Male	Cuff catheter	Right internal jugular vein	Damage to the right main bronchus	Right side chest tube No. 32 Fr was inserted	Recover
Zhou et al. (1)	64	Female	Cuff catheter	Left internal jugular vein	Centra venous perforation	Using coils and cyanoacrylate glue	Recover
Wang et al. (2)	78	Female	Temporary catheter	Right subclavian vein	Penetration wound the superior vena cava	Opening chest and surgical repair	Recover

In this kind of complication, direct removal of a catheter is very dangerous after vascular injury complications occur, and such complications can lead to a large mediastinum hematoma and even threaten the life of a patient if the catheter damages an artery. Previous case reports of catheter injuries to the central vein indicate that various measures can be effective at different locations (6, 7, 9). Siordia et al. (8) reported that the mini-sternotomy approach provided sufficient visualization of the vessel and surrounding structures, with minimal post-operative complications and healing time. This new approach replaced the traditional complete median sternotomy in attempts to repair the innominate vein. In the current case, thoracotomy was deemed unfeasible, because the patient was suffering from anemia and uremia and may not have tolerated the associated trauma. The cost of such an operation would also have been high. In another report, DSA-guided direct injection of coils and cyanoacrylate glue into the sinus tract was used to treat central venous perforation that occurred during placement of a central venous catheter (1). These methods were used to treat serious cases however, in which the patients had developed pericardial effusion and hemorrhagic shock. In the present patient, although brachiocephalic vein perforation occurred, there was no hematoma, and he did not report pain or discomfort. The vascular breach of the brachiocephalic vein was able to be repaired with just 2 embolization microcoils. In the present case, the method was feasible and safe. During the operation, the patient did not report any substantial discomfort. He was discharged the day after the surgery. Then, he was followed up for 1 month after surgery. The related complications, such as bleeding, infections, falling off the embolization microcoils, and thrombosis of injured blood vessels, did not occur (as shown in Figure 3B), and he is currently well and continues to be treated *via* the left cuff catheter in a hemodialysis room.

Of note, although the method of embolization microcoil for the repair of brachiocephalic vein perforation is safe and effective, some complications of using embolization microcoils should be paid attention. 1 is to short-term complication that the embolization microcoil falls off during operation. A senior interventional doctor is the best candidate if the operation of using embolization microcoil must be performed. The other is to long-term complication that thrombosis of injured blood vessels occurred after surgery. Use of heparin every other day can prevent the thrombosis of injured blood vessels, and patients who need to continue to treat with hemodialysis may ignore this note. On the contrary, patients who do not need to continue to treat with hemodialysis need anticoagulant to prevent the thrombosis after the embolization microcoil operation. Lastly, the specific usage of anticoagulant needs to be adjusted according to the patient's coagulation function.

## Conclusion

Brachiocephalic vein perforation during hemodialysis catheter placement is rare. Checking the location of the hemodialysis catheter is very important after catheter placement is accomplished. DSA-guided placement of a hemodialysis catheter can reduce the risk of serious complications and secondary damage. Consultation with multidisciplinary experts is necessary to reduce the risk of serious complications arising from catheter placement. Lastly, embolization microcoil blocking of brachiocephalic vein perforation is effective and safe.

## Data Availability Statement

The original contributions presented in the study are included in the article/supplementary material, further inquiries can be directed to the corresponding author/s.

## Ethics Statement

The studies involving human participants were reviewed and approved by The Ethical Committee of the People's Hospital of GuiZhou Province. The patients/participants provided their written informed consent to participate in this study. Written informed consent was obtained from the individual(s) for the publication of any potentially identifiable images or data included in this article.

## Consent for Publication

The patient whose case is described in this report has provided written informed consent for its publication.

## Author Contributions

PF and YZ conceived the idea of this article. YH, MS, and LM collected the data and wrote the manuscript. FG and PY contributed to the data collection and monitored the patient in the clinic. All authors read and approved the final manuscript.

## Funding

This work was supported by grants from the National Natural Science Foundation of China (81760125), the Guizhou Science and Technology Support Plan Project (Guizhou Science and Technology Support Project [2019] No. 2801), and the NHC Key Laboratory of Pulmonary Immunological Diseases (Guizhou Provincial People's Hospital, Guiyang, Guizhou, China).

## Conflict of Interest

The authors declare that the research was conducted in the absence of any commercial or financial relationships that could be construed as a potential conflict of interest.

## Publisher's Note

All claims expressed in this article are solely those of the authors and do not necessarily represent those of their affiliated organizations, or those of the publisher, the editors and the reviewers. Any product that may be evaluated in this article, or claim that may be made by its manufacturer, is not guaranteed or endorsed by the publisher.
